# A comparison of vulnerability factors in patients with persistent and remitting lifetime symptom course of depression^☆^

**DOI:** 10.1016/j.jad.2013.09.001

**Published:** 2014-01

**Authors:** Thorsten Barnhofer, Kate Brennan, Catherine Crane, Danielle Duggan, J. Mark G. Williams

**Affiliations:** a University of Oxford, Oxford Mindfulness Centre, Warneford Hospital, Oxford, OX3 7JX, UK; b Freie Universitaet Berlin, Dahlem Institute for Neuroimaging of Emotions, 14195 Berlin, Germany

**Keywords:** Depression, Lifetime course, Chronicity, Experiental avoidance, Childhood adversity

## Abstract

**Background:**

Research has suggested fundamental differences between patients with persistent and those with remitting courses of depression. This study investigated whether patients with different lifetime symptom course configurations differ in early risk and cognitive vulnerability factors.

**Methods:**

Patients with at least three previous episodes who were currently in remission were categorized based on visual timelines of their lifetime symptom course and compared with regard to a number of different indicators of vulnerability including questionnaire measures of childhood trauma and experiential avoidance.

**Results:**

Of the *N*=127 patients, *n*=47 showed a persistent course of the disorder with unstable remissions and symptoms most of the time, and *n*=59 showed a course with more stable, lasting remissions. Group comparisons indicated that patients with a more persistent course were significantly more likely to have suffered from childhood emotional abuse, and reported higher levels of experiential avoidance as well as related core beliefs. Experiential avoidance partially mediated the effect of childhood emotional abuse on persistence of symptoms.

**Limitations:**

The study is cross-sectional and does not allow conclusions with regard to whether differentiating variables are causally related to chronicity. Self-report measures may be subject to reporting biases.

**Conclusions:**

The results highlight the detrimental effects of childhood adversity and suggest that experiential avoidance may play an important role in mediating such effects.

## 1 Introduction

In many patients, depression takes a protracted course, in which symptoms persist or frequently recur, with the most common course characterized by fluctuation between symptoms on levels of full episodes or residual symptoms and times of relative recovery (Judd and Akiskal, 2000; Kennedy et al., 2004). While classification systems differentiate a number of different configurations, it has been suggested that the course of depression may most parsimoniously be described on a continuum of chronicity that takes into account residual levels of symptoms and is relatively orthogonal to depression severity (Klein, 2008; Torpey and Klein, 2008). Consistent with this assumption research has demonstrated fundamental differences between patients with a more persistent course, in which remissions often remain unstable, and patients with a more episodic course, in which remissions are more stable and lasting. However, little is currently known about psychological mechanisms underlying this differentiation. The main aim of the current research was, therefore, to investigate differences in cognitive vulnerability factors between these two groups. Chronic or persistent forms of depression represent a considerable challenge for established treatments (Cuijpers et al., 2010), and knowing about such differences is an important prerequisite for the development of more effective treatments.

There are a number of findings that support the differentiation of patients with more chronic and episodic courses. Compared to those with an episodic course of depression, patients with a more chronic course of the disorder have been found to show higher familiality, are more likely to have suffered from childhood adversity, are characterized by higher levels of temperamental vulnerability, e.g. neuroticism and introversion, are more likely to have suffered from co-morbid anxiety, substance abuse, and personality disorders, in particular of the avoidant type. Furthermore, patients with a chronic course of the disorder are more likely to have suffered from suicidality as part of their depression (for an overview see the meta-analysis by Hölzel et al., 2011). At the same time, research comparing different course configurations of chronic forms of depression, i.e. chronic depression, dysthymia, double depression, has found little differences in terms of demographic variables, symptom patterns, treatment response or family history (Klein et al., 2004; McCullough et al., 2003, 2000; Yang and Dunner, 2001). Comparing different definitions of chronicity, Mondimore et al. (2007) found that use of a broader definition in terms of ratings of the lifetime symptom course of the disorder, i.e. differentiating between patients who had suffered from symptoms most or all of the time versus those who had experienced lasting remissions, produced more pronounced differences in familiality than categorization based on DSM-IV criteria of chronic depression. The current study followed these findings by adopting a lifetime symptom perspective.

What are the cognitive factors that differentiate those who develop a more persistent course from those who achieve lasting remissions? Most of the factors listed above are either historical or relate to patients' current or past psychopathology, and few studies to date have investigated factors that might provide further information about cognitive mechanisms. This is surprising given the large body of cognitive research that has elucidated the role of depressive thinking in the maintenance of symptoms, both in terms of its content and process characteristics. On a content level, cognitive research has highlighted the role of enduring dysfunctional beliefs (Kovacs and Beck, 1978) and early maladaptive schemata (Young, 1995), with the latter assumed to represent a broader set of themes that have their origin in childhood. On a process level, research has demonstrated how maladaptive responses to negative mood such as rumination (Nolen-Hoeksema, 1991), suppression (Wenzlaff and Luxton, 2003), and experiential avoidance (Hayes et al., 1996) play an important role in maintaining negative mood with research on rumination also demonstrating effects on length of depressive episodes (Nolen-Hoeksema, 2000). Rumination undermines active attempts at problem-solving, reinforces negative biases and dysfunctional attitudes, and, in line with the idea that the above processes can be considered facets of a more encompassing maladaptive mode of processing (Williams, 2008), has been suggested to serve an avoidant function (Nolen-Hoeksema et al., 2008). Furthermore, research shows that depressed patients tend to frame their experience and thoughts in abstract and general terms. Autobiographical memory overgenerality has been found to be related to history of childhood adversity and abuse, rumination, deficits in interpersonal problem solving, and has been demonstrated to be a significant predictor of time to recovery in those who are currently depressed (for an overview see Williams et al., 2007).

While these cognitive factors are clearly implicated in maintenance of negative mood, there are currently few studies that have investigated the extent to which these are distinguishing characteristics of patients with more or less persistent courses of depression. Riso et al. (2003) compared patients with chronic depression to patients with non-chronic Major Depression and found that, after controlling for current levels of depression, patients with chronic depression showed higher levels of maladaptive core beliefs relating to the themes of disconnection and rejection, impaired autonomy and overvigilance. However, there were no significant differences in ruminative tendencies, attributional style or dysfunctional attitudes, suggesting that chronically depressed patients may differ from others predominantly with regard to factors with a stronger developmental origin. In contrast, a more recent and larger study comparing patients with chronic and non-chronic depression categorized based on the course of the disorder during the last five years (Wiersma et al., 2011) found that chronically depressed patients reported significantly stronger tendencies to respond with ruminative thinking during times when they are feeling low. Additionally, chronically depressed patients showed lower levels of extraversion and higher external locus of control.

One of the reasons for these inconsistencies might have been that these studies investigated patients who were currently suffering from high levels of symptoms. Rumination, memory overgenerality, dysfunctional attitudes and other cognitive vulnerability factors are all positively related to levels of symptoms and the resulting state-related elevation in maladaptive cognitive characteristics may have obscured more lasting differences that may become visible when comparing groups with lower levels of symptoms. A second reason for uncertain results is the use of relatively short timelines to judge the pattern of chronicity.

The current study differed from previous research by testing patients at a time when they were in remission and differentiating them based on their lifetime symptom history. We assessed a sample of patients, recruited for a trial of Mindfulness-Based Cognitive Therapy for relapse prevention, who had a high risk of relapse and a prolonged history of depression, but were in recovery at entry into the trial and at the time of assessment. A previous trial of MBCT that followed participants with similar characteristics prospectively after they had received therapy had found that participants could be meaningfully divided into about equally large groups of patients with stable and unstable remission (Segal et al., 2010). We classified participants with regard to whether they showed a lifetime symptom course that was characterized by good and sustained remissions between episodes or a more persistent course of the disorder where remissions remained unstable, following the procedure developed by Mondimore et al. (2007). Our main aim was to see whether the two groups differed with regard to content and process characteristics of depressive thinking including overgeneral memory, ruminative tendencies, experiential avoidance, dysfunctional attitudes, and core beliefs related to suicidality. We also tested whether the two groups differed with regard to childhood adversity. Furthermore, given previous findings pointing towards developmental origins of cognitive differences, we were interested to explore whether there was evidence for effects of childhood adversity to be mediated through any of the cognitive factors assessed in our study.

## 2 Method

### 2.1 Participants

The current sample consisted of participants who were recruited to take part in the Oxford-arm of the Staying Well after Depression-Trial, a multi-center randomized-controlled trial of treatments aimed at reducing risk for relapse to depression. Participants were included in the study if they (a) had a history of three or more major depressive episodes according to DSM-IV-TR criteria in the past, two of which had to have occurred in the past five years and one in the past two years, (b) were currently in recovery, which was defined as not having experienced more than one symptom of depression for more than a week over the last eight weeks, (c) were not currently suffering from an eating disorder or an obsessive-compulsive disorder, had not suffered from bipolar disorder or schizophrenia, and were not having significant problems with substance dependence or substance abuse, (d) were not regularly self-harming, (e) were between 18 and 70 years of age, and (f) were fluent in spoken and written English. Individuals interested in the trial had either made contact with the research team on their own initiative after having heard or read about the study through media advertisements or had been referred through their GPs. First contacts occurred on the phone where potential participants were screened for main inclusion and exclusion criteria. Those who seemed eligible were invited to come to the Department of Psychiatry for a diagnostic session that included a full Structured Clinical Interview for DSM-IV TM (First et al., 2002) conducted by trained research psychologists in the context of which the lifetime course of depression was assessed using timelines with continuous mood ratings on a visual analog scale. 152 individuals participated in the assessments, of whom *n*=127 provided sufficiently precise information for a timeline to be completed.

### 2.2 Interviews and questionnaires

#### 2.2.1 Structured Clinical Interview for DSM-IV (SCID) and visual timeline

Current and past diagnostic status was assessed using the Structured Clinical Interview for DSM-IV (First et al., 2002) conducted by trained research psychologists. In order to assess lifetime history of depression, interviewers used a visual timeline with age depicted on the *x*-axis and level of depression on the *y*-axis. Auxiliary lines on the *x*-axis indicated the beginning and end of each year from age 10 to 70, auxiliary lines on the *y*-axis indicated the extreme points of worst and best mood and the zero point of the dimension. Participants were first asked to indicate on the timeline a number of anchor points that reflected important events or periods in their lives such as the time they had lived in a particular city, the beginning and end of their school years, or further education, the time they had been in a particular job, marriage, the birth of children or other events that individuals felt were important. They were then asked to mark with a glue dot the worst point of each episode of depression that they had reported in the SCID interview. In a next step, participants connected the dots by indicating how levels of depression changed over time. Participants were asked to use the zero-point of the scale as a reference point indicating normal mood without any symptoms of depression and to sketch out changes in depression levels over time indicating both mild and severe levels of depression as well as variations in positive mood states.

#### 2.2.2 Ratings of chronicity

Global ratings of chronicity of the lifetime course were derived from the information given by the visual timelines, made by an assessor that was blind to other characteristics of the patient. Times at which ratings were at or above the zero-point of the depression scale were taken as episodes of recovery while times during which depression ratings were at or around low points, which SCID interviews ascertained to be indicative of full episodes, were taken to represent length of full episodes. Depression ratings that were between levels of full episodes and recovery were taken to indicate times at which patients suffered from residual or minor levels of symptoms. Lifetime course of depression since first onset was rated by two independent raters as 1=“remitting” (“good remissions substantially longer than episodes”), 2=“frequent/brief episodes (<3 weeks) without prolonged remissions”, 3=“double or chronic” (“substantial mood symptoms most or all of the time”) or 4=“other” using the scale by Mondimore et al. (2007).

Agreement between the two raters was determined using weighted Kappa and found to be at an acceptable level, *κ*=.71, *p*<.001. In cases of disagreement, the raters discussed the rating to come to a consensual decision.

#### 2.2.3 Beck Depression Inventory II (BDI-II) (Beck et al., 1996)

The BDI-II is a widely used self-report questionnaire for the assessment of the severity of current symptoms of depression. The BDI-II contains twenty-one statements, assessing symptoms over the preceding two weeks. Internal consistency in the current sample was *α*=88.

#### 2.2.4 Beck Hopelessness Scale (BHS) (Beck, 1988)

The BHS contains 20 statements describing negative and positive attitudes towards the future. Internal consistency in our sample was *α*=.87.

#### 2.2.5 GAD-7 Anxiety Scale (Spitzer et al., 2006)

The GAD-7 is a brief measure for assessing symptoms of Generalized Anxiety Disorder. The questionnaire contains 7 items assessing severity of generalized anxiety symptoms over the preceding 2 weeks. Internal consistency in our sample was *α*=.84.

#### 2.2.6 Autobiographical Memory Task (AMT) (Williams and Broadbent, 1986)

Participants were presented with 18 cue words (9 positive, and 9 negative trait adjectives) matched for frequency, emotionality and imageability (Barnhofer et al., 2007) and instructed to recall a specific memory in response to each cue. A specific memory was defined as a memory referring to an event that occurred at a particular time and place and lasted no longer than 1 day. Participants were instructed not to repeat memories. Examples were given and participants completed 3 practice items. They were given 30 s to begin their response for each cue during test phase. All responses were audio-taped and scored by the experimenter for level of specificity as either specific, extended, categoric, semantic associations, or omissions. In line with general practice we used the number of specific memories as the main outcome measure.

#### 2.2.7 Childhood Trauma Questionnaire (CTQ) (Bernstein and Fink, 1998)

The Childhood Trauma Questionnaire is a 28-item self-report inventory designed to provide a screening for histories of abuse and neglect. Subscales assess five different types of maltreatment – emotional, physical, and sexual abuse, and emotional and physical neglect. The questionnaire also includes a 3 item minimiziation/denial scale for detecting false-negative trauma reports. Internal consistencies of the subscales in the current sample reached from *α*=.72 to .95 (emotional abuse =.89; physical abuse =.86; sexual abuse =.95; emotional neglect =.93; physical neglect =.72).

#### 2.2.8 Acceptance and Action Questionnaire (AAQ) (Hayes et al., 2004)

The AAQ is a brief self-report measure for assessing key aspects of experiential avoidance such as inaction, literalness of thoughts, controlling of internal events, and escape of negative content. The 10-item version was used. Internal consistency in the current sample was *α*=.87.

#### 2.2.9 Ruminative Response Style Questionnaire (RRSQ) (Treynor et al., 2003)

The RRSQ assesses the extent to which individuals respond to depressed mood by focusing on self, symptoms and the possible causes and consequences of their mood. The questionnaire contains 22 items. Treynor et al. (2003) differentiate three subscales: depression-related rumination (analytical rumination focused on the symptoms of depression, “Think about how sad you feel”), brooding (described as “moody pondering”, Think “Why can’t I handle things better?”) and reflection (which relates to a more neutral form of pondering, “Analyze recent events to try to understand why you are feeling depressed”). Internal consistencies in our sample were *α*=.86 for depression-related rumination, *α*=.73 for brooding, and *α*=.69 for reflection.

#### 2.2.10 Dysfunctional Attitudes Scale (DAS) (Weissmann, 1979)

The DAS is a self-report inventory designed to assess the endorsement of dysfunctional beliefs guiding an individual's self-evaluation. Form A of the questionnaire, which was used here has been found to consist of two factors: “performance evaluation”, which refers to contingencies of self-worth and achievement (“If I do not do as well as other people, it means I am an inferior human being”), and “approval by others”, which refers to attitudes relating self-worth and social success (“My value as a person depends greatly on what others think of me”). The DAS Form A contains 40 items. Internal consistency in our sample was *α*=.93.

#### 2.2.11 Suicidal Cognitions Scale (SCS) (Rudd et al., 2001)

The SCS assesses cognitive dimensions of suicide-specific hopelessness including perceived burdensomeness (“I am a burden to my family”), helplessness (“No one can help solve my problems”), unlovability (“I am completely unworthy of love”) and poor distress tolerance (“When I get this upset, it is unbearable”). The scale comprises 20 items. Internal consistencies in the current sample were *α*=.49 for perceived burdensomeness, *α*=.80 for unlovability, *α*=.81 for helplessness, and *α*=.90 for distress tolerance.

### 2.3 Procedure

Participants were first invited to take part in a diagnostic session at the Department of Psychiatry during which the structured clinical interview was conducted and participants also filled in several self-report questionnaires including the BDI-II. In the second session, participants completed a number of cognitive tests of which only the AMT is relevant to the current analyses. All participants had given their informed consent and the whole study had received ethical approval from the National Research Ethics Service (Oxfordshire Rec C) (MREC 08/H0606/56).

## 3 Results

### 3.1 Classification of participants

Consensual ratings on the Mondimore scale classified *n*=59 (46%) participants as showing a remitting lifetime course, *n*=2 (1%) showing a frequent/brief episodes without prolonged remissions, *n*=47 (37%) showing a chronic lifetime course, and *n*=19 (14%) as other. Further analyses focussed on the comparison of participants with remitting and persistent lifetime course only.

### 3.2 Group comparisons

In a first step we tested group differences with multiple univariate tests using ANOVAs for continuous variables and *χ*^2^-tests for categorical variables. Results of these analyses and the respective descriptive statistics are listed in Table 1. There were no significant differences between the two groups in baseline characteristics including socio-demographic variables, current levels of symptoms, co-morbid anxiety disorders, age of onset and current use of antidepressants apart from the finding that a significantly higher number of patients with persistent depression reported a history of substance abuse or dependence.

Analyses of differences in early risk factors and psychological variables showed significantly higher levels of childhood adversity in those with a persistent course with significant differences emerging on the CTQ scales of Emotional Abuse and Emotional Neglect. Furthermore, compared to those with a remitting course, participants with a persistent course showed significantly lower scores on the AAQ, i.e. higher levels of avoidance, and significantly higher scores on the Suicide Cognitions Helplessness sub-scale, as well as significantly lower scores on the Distress Tolerance sub-scale. There were no significant differences between the two groups in dysfunctional attitudes as measured by the DAS, and in ruminative tendencies as measured by the RRSQ. There were also no significant differences in autobiographical memory specificity. Results remained virtually unchanged when they were adjusted for the effect of age, gender, current symptoms of depression and anxiety, and history of substance abuse and dependence, with the exception of the total score and helplessness scale of the Suicide Cognitions Scale, which were reduced to trend levels (*p*=.05). Significance levels of the adjusted tests are listed in Table 1.

In order to compare the magnitude of risk for persistence associated with the early risk factors and psychological variables we computed adjusted odds ratios using multiple logistic regressions controlling for the effect of age, gender, current symptoms and history of substance abuse or dependence. The size of the OR was generally small: OR=1.11; 95% CI [1.02, 1.20] for CTQ Emotional Abuse, 1.08; [1.01, 1.16] for Emotional Neglect,.93; [.88,.98] for the AAQ, and 1.13; [1.02, 1.26] for SCS Distress Tolerance.

In a stepwise multiple logistic regression, in which age, gender, current symptoms, early risk factors and previous history of disorders were entered in the first step, and psychological variables were entered in the second, CTQ Emotional Abuse emerged as a significant factor, *B*=.17, *SE*=.08, *Wald*=4.23, *df*=1, *p*=.04, in the first step. In the second step, with all variables entered into the equation the AAQ sumscore, *B*=−.11, *SE*=.05, *Wald*=5.36, *df*=1, *p*=.02, emerged as a significant predictor, while CTQ Emotional Abuse was reduced to be only marginally significant suggesting that the effect of childhood emotional abuse was mediated through experiential avoidance.

### 3.3 Mediational analyses

In order to more formally test this assumption, we computed direct and indirect effects of childhood emotional abuse on the course of the disorder (remitting versus persistent) using logistic regressions and applying the bootstrapping approach by Preacher and Hayes (2004). This approach allows direct significance testing of the indirect effect of the independent variable on the dependent variable through the mediator quantified as the product of the effect of the independent variable on the mediator, *a*, and the effect of the mediator on the dependent variable, partialling out the effect of the independent variable, *b*. A point estimate of the indirect effect was derived from the mean of 5000 estimates of *a*×*b* and 95% percentile-based confidence intervals were computed using the cut-offs for the 2.5% highest and lowest scores of the empirical distribution. An indirect effect is considered significant when the bias corrected and accelerated confidence interval does not include zero. There was a significant indirect effect, *a*×*b*=.02; 95% CI [.002–.064], estimated as the product of the path from the independent variable (CTQ emotional abuse) to the mediator (AAQ sumscore), *a*=−.36, *SE*=.15, *t*=−2.37, *p*=.02, and from the mediator to the dependent variable (course of the disorder), *b*=−.06, *SE*=.02, *Wald*=4.91, *p*=.03, and only a marginally significant direct effect of CTQ Emotional Abuse on the course of the disorder *c*′=.077, *SE* =.04, *Wald*=3.56, *p*=.06, thus supporting the assumption of a mediational effect of experiential avoidance.

## 4 Discussion

Few studies have investigated differences in psychological variables between patients for whom depression takes a course with good remissions, compared with those for whom it is more chronic and persistent. The current research adopted a broader lifetime perspective differentiating between patients with lifetime symptom courses that were characterized by the persistence of symptoms for most of the time since onset of the disorder or by full and lasting remissions between episodes. We assessed patients at a point when they were in recovery. Despite the fact that the current sample was highly homogeneous with regard to prior number of depressive episodes and their pattern – two of the previous episodes had to have occurred in the past five years and one in the past two years – a considerable proportion of the participants, 83%, could be classified in this way with satisfactory inter-rater reliability. This is consistent with other recent research that has found that about half of the patients with three or more previous episodes do not reach stable remissions following a full course of treatment (Segal et al., 2010), and suggests that differentiation of lifetime symptom courses with regard to the stability of remission may meaningfully add to the characterization of the course of depression.

Comparisons of the two groups suggest a number of distinguishing characteristics. Firstly, patients with a persistent course reported significantly higher levels of childhood adversity. This is consistent with previous research using definitions of chronicity according to current classification systems (Lizardi et al., 1995), and in line with a considerable body of research showing that childhood adversity is associated with enduring cognitive and biological vulnerabilities for depression (Danese and McEwen, 2012). In comparison to those with a remitting course, patients with a persistent course reported higher levels of emotional abuse and emotional neglect from their caregivers with emotional abuse emerging as the overarching factor that rendered effects of the related, but more specific (Baker and Festinger, 2011), neglect factor non-significant when entered in analyses simultaneously. While there were significant relations between persistence and history of emotional abuse, we neither found a relation between persistence and physical abuse or neglect, nor between persistence and sexual abuse, which is in contrast to other findings (Fogarty et al., 2008; McNally et al., 2006). Because levels of physical abuse and neglect, and sexual abuse seemed generally lower than levels of emotional abuse and neglect it might have been more difficult in our study to detect such relations.

Secondly, patients with a persistent lifetime course were characterized by significantly higher levels of experiential avoidance, helplessness, and distress intolerance. This is in line with previous research that has established experiential avoidance as a general risk factor for psychopathology (Chawla and Ostafin, 2007) and highlights the importance of patients' responses to negative internal events for the course of the disorder. The results suggest that tendencies to respond to negative internal events with avoidance and withdrawal, low tolerance for such events and a tendency to respond with helplessness rather than active attempts at coping represent a significant risk for chronicity. Experiential avoidance as assessed by the AAQ represents a complex construct that covers a range of different facets including beliefs about emotions, avoidant behaviors, fear of emotions, and cognitive responses related to avoidance such as worry. The fact that the effects of SCS Helplessness and Distress Tolerance were rendered non-significant when experiential avoidance was considered at the same time suggests that they reflect facets subserving or arising from the broader construct of experiential avoidance.

In the light of the concept of a core process encompassing experiential avoidance and rumination, it is surprising that there was no group difference in rumination. This finding is consistent with results from the study by Riso et al. (2003), who, after controlling for current level of depression, also failed to find a difference in rumination, but inconsistent with findings from the study by Wiersma et al. (2011) where rumination emerged as of one the main differentiating factors. Given that the study by Wiersma et al. was based on a much larger sample, it is most likely that inconsistencies are simply due to failure to detect existing differences in the other two studies. However, it is interesting to note that the study by Riso et al. and our study both used the RRSQ to measure rumination, whereas Wiersma et al. used the Leiden Inventory of Depression Sensitivity (LEIDS) (Van der Does, 2002), which is a measure of cognitive reactivity. There remains a possibility, therefore, that patients with persistent and remitting lifetime course may not so much differ in general levels of rumination, but more with regard to the ease with which such a response can be triggered through only minor events such as changes in mood. The two groups also did not differ in levels of autobiographical memory specificity, which is unexpected, particularly because overgenerality of autobiographical memory has previously been related to childhood adversity and avoidant tendencies (Williams et al., 2007). There is considerable evidence that overgenerality is an important predictor of the course of depression (Sumner et al., 2010), yet the current data do not support the idea that it is a distinguishing characteristic of patients with a persistent lifetime course. A possible explanation for this discrepancy might be that previous research demonstrating relations between overgenerality and persistence of depressive symptoms has usually assessed memory specificity during episode, and it is possible that assessment of overgenerality during times of recovery may not have similar predictive power as deficits are likely to be reduced.

In addition to the above process characteristics we also tested differences in content characteristics by comparing the two groups with regard to their endorsement of dysfunctional attitudes. The failure to find any significant differences in dysfunctional attitudes between the two groups parallels findings by Riso et al. (2003) who found that, after controlling for current level of depression, groups differed in schemata but not in dysfunctional attitudes, suggesting that relevant differences are more likely to reside on a level that is likely to have a stronger developmental origin and combines affective and cognitive components to a greater degree, which is consistent with our findings regarding the role of early emotional abuse.

Investigation of mediational pathways showed that the effect of childhood emotional abuse on persistence was mediated through experiential avoidance. While the fact that the current study was cross-sectional restricts conclusions regarding temporal order, these findings are in line with research showing that early adversity has profound and lasting effects on individuals' stress responses (Danese and McEwen, 2012). Experiential avoidance, decreased distress tolerance, increased tendency to respond with helplessness are likely psychological consequences of early adversity and concomitants of such changes, and according to our findings, play a significant role in whether patients suffer persistent courses of depression or achieve good remissions.

## 5 Limitations

The current study has a number of limitations. Firstly, the study is cross-sectional and therefore does not allow any conclusions with regard to whether the psychological variables that differentiate between groups are causally related to persistence. Secondly, the current data are all based on self-report or interview and thus may be subject to reporting biases although the fact that participants were assessed while they were in recovery reduced the scope for possible mood-related biases. Thirdly, the study is based on a relatively small number of patients and is therefore in need of replication. Fourthly, all of the participants had to agree to participate in a clinical trial and inclusion criteria for this trial were relatively narrow, i.e. history of at least three previous episodes of depression, which might have compromised representativeness of the sample.

## 6 Summary and conclusions

In summary, this study highlights experiential avoidance and related characteristics of an avoidant style such as helplessness and low distress tolerance as distinguishing characteristics of patients with a persistent lifetime symptoms course, and suggests that these characteristics may be particularly likely to arise in those with and as a consequence of early emotional abuse. Treatment of chronic depression still represents a considerable challenge (Cuijpers et al., 2010) and there is evidence that currently established treatments are significantly less effective in patients with a history of childhood adversity (Nanni et al., 2012). Interventions that target avoidance of internal events such as mindfulness interventions, which have produced promising preliminary evidence in chronic depression (Barnhofer et al., 2009), and interventions that target avoidance of external events such as behavioral activation (Dimidjian et al., 2011), which has not yet been systematically investigated for use in persistent depression, may be particularly helpful in this context.

## Role of funding source

This study was funded by Grant GR067797 from the Wellcome Trust to J.M.G. Williams. The funders had no role in study design, data collection and analysis, decision to publish, or preparation of the manuscript.

## Conflict of interest

*Disclosure of interest*: The authors declare they have no conflicts of interest with regard to this paper.

## Figures and Tables

**Table 1:** Baseline demographics, clinical and cognitive characteristics of participants with persistent (*n*=47) and remitting (*n*=59) course of MDD.

Characteristic	Groups		Analysis			
	Chronic	Remitting	Test statistic	*df*	Unadjusted *p*	
Age in years, *M* (*SD*)	44.00 (11.30)	41.20 (12.70)	*F*=1.39	1	.24	
Gender, n female (%)	33 (73)	42 (71)	*χ*^2^=.01	1	.81	
Age of onset, *M* (*SD*)	18.51 (9.36)	21.05 (11.22)	*F*=1.54	1	.21	
Relationship status						
Currently in relationship, *n* (%)	26 (56)	38 (66)	*χ*^2^=.87	1	.34	
Currently not in relationship, *n* (%)	20 (44)	20 (34)				
Ethnicity						
Caucasian-white, *n* (%)	4 (96)	56 (94)	*χ*^2^=.03	1	.86	
Other, *n* (%)	3 (4)	2 (6)				
Beck Depression Inventory, *M* (*SD*)	7.74 (8.18)	7.40 (6.73)	*F*=.05	1	.81	
Beck Hopelessness Scale, *M* (*SD*)	5.17 (4.80)	4.20 (3.64)	*F*=1.33	1	.25	
GAD7 – Anxiety scale, *M* (*SD*)	2.23 (3.02)	2.31 (2.95)	*F*=.02	1	.89	
Current anxiety disorder, *n* (%)	7 (14)	10 (17)	*χ*^2^=.08	1	.77	
History of anxiety disorder, *n* (%)	18 (39)	22 (37)	*χ*^2^=.01	1	.99	
History of substance dependence or abuse, *n* (%)	10 (21)	3 (5)	*χ*^2^=6.37	1	.01	
Currently taking antidepressants, *n* (%)	18 (46)	22 (42)	*χ*^2^=.23	1	.63	

Autobiographical memory test			Test statistic	*df*	Unadjusted *p*	Adjusted *p*^a^

Specific Memories, *M* (*SD*)	9.55 (4.31)	9.77 (3.47)	*F*=.09	1	.76	.72
Childhood trauma questionnaire						
Emotional abuse, *M* (*SD*)	11.95 (5.87)	9.10 (4.72)	*F*=7.70	1	.01	.01
Physical abuse, *M* (*SD*)	8.12 (4.89)	6.72 (3.70)	*F*=2.80	1	.10	.27
Sexual abuse, *M* (*SD*)	6.12 (3.84)	7.15 (4.76)	*F*=1.43	1	.23	.13
Emotional neglect, *M* (*SD*)	13.27 (6.33)	10.93 (5.14)	*F*=4.41	1	.03	.04
Physical neglect, *M* (*SD*)	7.74 (3.67)	6.64 (2.73)	*F*=3.12	1	.08	.26
Action and acceptance questionnaire						
Total Score, *M* (*SD*)	37.26 (8.43)	41.86 (8.68)	*F*=7.79	1	.01	.01
Ruminative response style questionnaire						
Total score, *M* (*SD*)	56.55 (10.88)	58.76 (10.72)	*F*=1.09	1	.29	.43
Depressive rumination, *M* (*SD*)	31.95 (6.97)	33.37 (6.67)	*F*=1.09	1	.29	.39
Reflection, *M* (*SD*)	11.93 (3.12)	12.50 (2.83)	*F*=.93	1	.33	.50
Brooding, *M* (*SD*)	12.95 (3.02)	13.25 (3.51)	*F*=.21	1	.64	.63
Dysfunctional attitudes questionnaire						
Total score, *M* (*SD*)	146.85 (38.49)	139.94 (29.18)	*F*=1.10	1	.29	.22
Performance evaluation, *M* (*SD*)	52.73 (18.51)	48.36 (14.82)	*F*=1.75	1	.18	.14
Need for approval, *M* (*SD*)	44.04 (10.79)	44.30 (8.12)	*F*=.02	1	.88	.96
Suicidal Cognitions Scale						
Total score, *M* (*SD*)	34.68 (14.73)	29.72 (8.78)	*F*=5.12	1	.03	.05
Perceived Burdensomeness, *M* (*SD*)	3.38 (1.66)	3.18 (1.13)	*F*=.52	1	.47	.37
Unloveability, *M* (*SD*)	9.74 (4.58)	8.64 (3.34)	*F*=2.04	1	.15	.28
Helplessness, *M* (*SD*)	9.53 (4.38)	7.98 (2.94)	*F*=4.70	1	.03	.05
Distress Intolerance, *M* (*SD*)	12.29 (6.01)	9.91 (3.46)	*F*=6.54	1	.01	.03

a Adjusted for age, gender, symptoms of depression (BDI-II) and anxiety (GAD-7), and history of substance dependence or abuse.
