# Supplementary material for: A comparison of vulnerability factors in patients with persistent and remitting lifetime symptom course of depression
Source: J Affect Disord. 2014 Jan;152-154:155–61. doi: 10.1016/j.jad.2013.09.001 (PMC3878770; doi:10.1016/j.jad.2013.09.001)
Supplement: Supplementary file 1 — Supplementary Material [file mmc1.pdf]

Supplemental Material

Table 1
Sequence of assessments to define groups of patients with persistent or remitting lifetime symptom course

Step	Instrument	Procedure	Main outcome variables
1.	SCID interview (First et al., 2002)	Assessment of current diagnostic status by trained psychologists to check inclusion and exclusion criteria. Assessors followed affective disorders module to verify remission, assess criteria for last episode of MDD, number and time of previous episodes of MDD and age of onset.	Number and time of previous episodes of MDD
2.	Visual timeline	Assessors use timeline together with participants to visualise symptom levels over entire lifetime. Participants were first asked to: indicate anchor points that reflect important events or periods in their lives, mark with a glue dot the worst point of each episode of MDD reported in the SCID interview, and then to connect dots to indicate how levels of depression had changed over time.	None (visualisation for later rating)
3.	Mondimore et al. (2006) scale for ratings of chronicity	Blind assessors make global ratings of chronicity/persistence based on timelines	Rating of lifetime symptom course as “remitting”, “frequent/brief episodes”, “double or chronic”, or “other”

Visual timeline examples

Figure 1 depicts the timeline of a fictional participant with a persistent course of the disorder (“double or chronic depression” on the Mondimore et al. scale). Anchor events inserted into the timeline include stressful events such as divorce of parents, relationship break-up, as well as anchors for particular periods of time such as the time at university, which were elicited to facilitate estimates for the beginning and

end of particular episodes of depression. After having indicated the timepoints at which the episodes of depression reported in the SCID interview had their worst point, participants were asked to join the dots that represented those worst points to produce a continuous timeline. The participant whose timeline is depicted in Figure 1 joined these point in a way that suggest that, although the participant experienced prolonged periods during which depression did not peak to the levels indicated for episodes of depression, symptoms were present continuously on a lower level and, since first onset, the participant never reached normal mood over prolonged periods of time. In contrast for the participant whose timeline is depicted in Figure 2, episodes of depression are clearly interspersed with episodes during which functioning and mood were on a normal level. The lifetime symptom course of this participant was therefore judged a “remitting”.

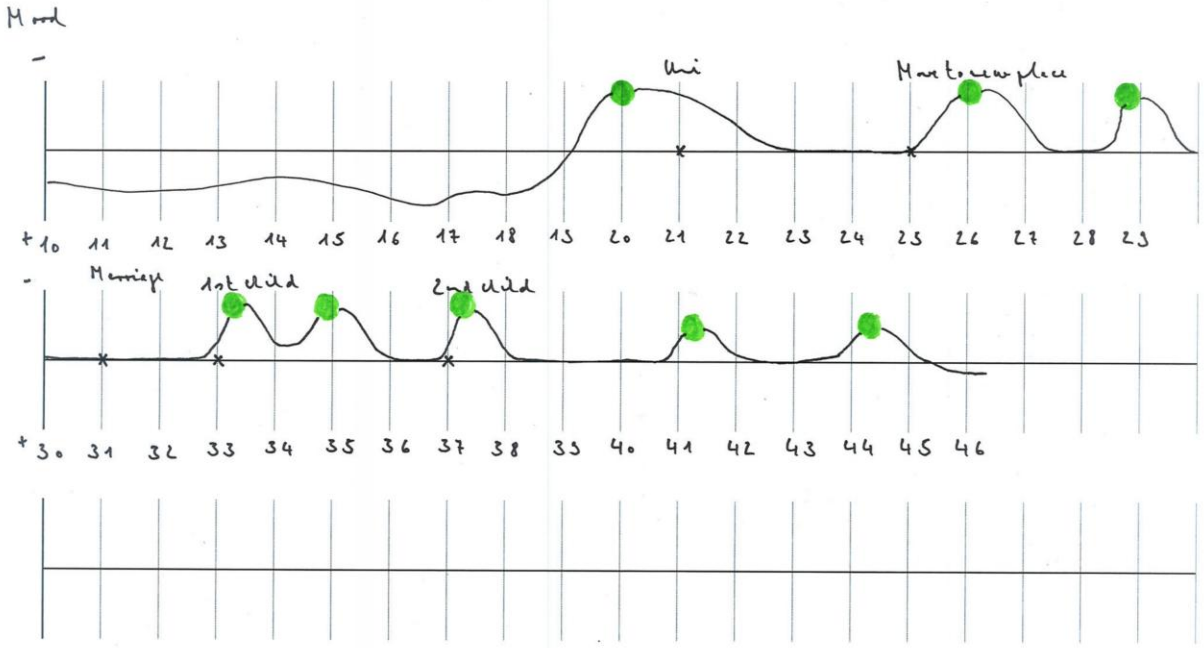

Figure 1. Timeline of a patient with persistent course of depression.

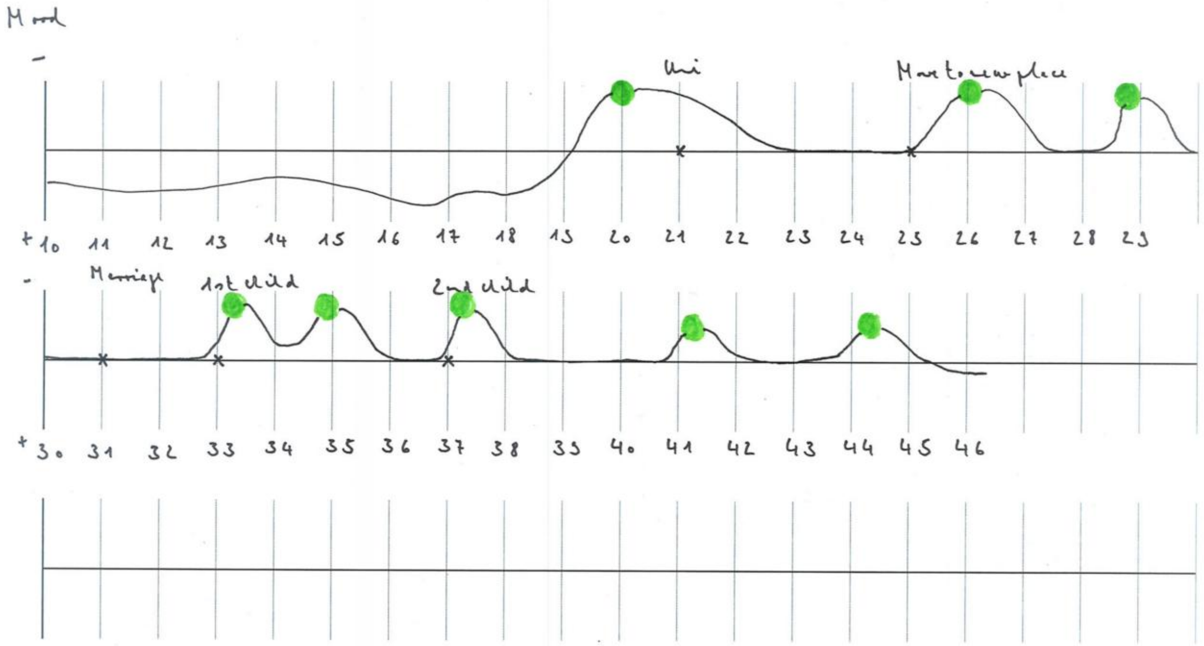

Figure 2. Timeline of a patient with remitting course of depression
